# Lag, lock, sync, slip: the many ‘phases’ of coupled flagella

**DOI:** 10.1098/rsif.2013.1160

**Published:** 2014-05-06

**Authors:** Kirsty Y. Wan, Kyriacos C. Leptos, Raymond E. Goldstein

**Affiliations:** Department of Applied Mathematics and Theoretical Physics, Centre for Mathematical Sciences, University of Cambridge, Wilberforce Road, Cambridge CB3 0WA, UK

**Keywords:** flagella, synchronization, *Chlamydomonas*

## Abstract

In a multitude of life's processes, cilia and flagella are found indispensable. Recently, the biflagellated chlorophyte alga *Chlamydomonas* has become a model organism for the study of ciliary motility and synchronization. Here, we use high-speed, high-resolution imaging of single pipette-held cells to quantify the rich dynamics exhibited by their flagella. Underlying this variability in behaviour are biological dissimilarities between the two flagella—termed *cis* and *trans*, with respect to a unique eyespot. With emphasis on the wild-type, we derive limit cycles and phase parametrizations for self-sustained flagellar oscillations from digitally tracked flagellar waveforms. Characterizing interflagellar *phase synchrony* via a simple model of coupled oscillators with noise, we find that during the canonical swimming breaststroke the *cis* flagellum is consistently *phase-lagged* relative to, while remaining robustly *phase-locked* with, the *trans* flagellum. Transient loss of synchrony, or *phase slippage*, may be triggered stochastically, in which the *trans* flagellum transitions to a second mode of beating with attenuated beat envelope and increased frequency. Further, exploiting this alga's ability for flagellar regeneration, we mechanically induced removal of one or the other flagellum of the same cell to reveal a striking disparity between the beatings of the *cis* and *trans* flagella, in isolation. These results are evaluated in the context of the dynamic coordination of *Chlamydomonas* flagella.

## 1. Introduction

Periodicity permeates Nature and its myriad life forms. Oscillatory motions lie at the heart of many important biological and physiological processes, spanning a vast dynamic range of spatial and temporal scales. These oscillations seldom occur in isolation; from the pumping of the human heart, to the pulsating electrical signals in our nervous systems, from the locomotive gaits of a quadruped, to cell cycles and circadian clocks, these different oscillators couple to entrain or are entrained by each other and by their surroundings. Uncovering the mechanisms and consequences of these entrainments provides vital insight into biological function. Often, it is to the aid of quantitative mathematical tools that we must turn for revealing analyses of these intricate physiological interactions.

**Figure 1.**
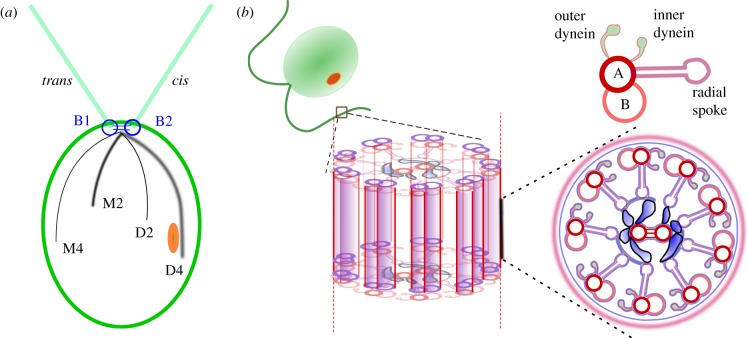
(*a*) Asymmetric cytoskeletal organization underlies beating differences between the two flagella of *C. reinhardtii*. A system of four acetylated microtubule rootlet bundles lies beneath the cell membrane, extending towards the cell posterior (four-membered rootlets: M4, D4; two-membered rootlets: M2, D2). During development, eyespot positioning delineates between the *cis* flagellum—closer to the eyespot, assembled from the daughter basal body (B2), and the *trans* flagellum—nucleated by the mother basal body (B1). (*b*) Inside the axoneme: a peripheral arrangement of microtubule doublets encircles a central pair, and specialized dynein motors initiate interdoublet sliding and beat generation.

The striking, periodic flagellar beats of one particular organism shall dictate the following discussion: *Chlamydomonas reinhardtii* is a unicellular alga whose twin flagella undergo bilateral beating to elicit breaststroke (BS) swimming. For these micrometre-sized cells, their motile appendages, termed flagella, are active filaments that are actuated by internal molecular motor proteins. Each full beat cycle comprises a *power stroke*, which generates forward propulsion, and a *recovery stroke* in which the flagella undergo greater curvature excursions, thereby overcoming reversibility of Stokes flows [1]. A single eyespot breaks cell bilateral symmetry, distinguishing the *cis* flagellum (closer to the eyespot) from the *trans* flagellum (figure 1). Subjected to internal modulation by the cell, and biochemical fluctuations in the environs, the two flagella undergo a rich variety of tactic behaviours. For its ease of cultivation and well-studied genotype, *Chlamydomonas* has become a model organism for biological studies of flagella/cilia-related mutations. For its simplistic cell–flagella configuration, *Chlamydomonas* has also emerged as an idealized system onto which more physical models of dynamic flagellar synchrony can be mapped [2–5]. With this versatility in mind, this article has two goals. First, we proffer a detailed exposition of *Chlamydomonas* flagella motion as captured experimentally by high-speed imaging of live cells; second, we develop a quantitative framework for interpreting these complex nonlinear motions.

In the light of previous work, we have found the motion of *Chlamydomonas* flagella to be sufficiently regular to warrant a low-dimensional phase-reduced description [2,3,6,7]. Single-flagellum limit cycles are derived from real time series and are associated with a phase (§3.2). For each cell, we formulate the dynamics of the flagella pair as mutually coupled phase oscillators (§3.3), whose pairwise interactions can be determined in our experiments to sub-beat-cycle resolution. Just as marching soldiers and Olympic hurdlers alike can have preferential footedness, we find that *Chlamydomonas* is no exception; resolving within each cycle of its characteristic BS gait we see that one flagellum is consistently phase-lagged with respect to the other. These transient episodes, previously termed *slips* [2], are to be identified with phase slips that occur when noisy fluctuations degrade the phase-locked synchronization of two weakly coupled oscillators of differing intrinsic frequencies [8]. Sampling multiple cells, each for over thousands of BS cycles, we clarify the non-constancy of synchrony over a typical cycle; in particular, the two flagella are found to be most robustly synchronized in the power stroke and least synchronized at the transition to the succeeding recovery stroke. This trend appears to be universal to all cells of the wild-type strain. As further indication that *Chlamydomonas* cells are highly sensitive to fluctuating biochemical cues, we find that the tendency for the two flagella of a given cell to experience phase slips exhibits much greater variation across the population (figure 6). Visualizing multiple phase-slip excursions via a dimension-reduced Poincaré return map of interflagellar phase difference, we demonstrate that the synchronized state is globally stable (figure 9). Examining each flagellar phase slip in detail we show further that the *trans* flagellum reproducibly transitions to a well-defined transient state with higher beat frequency and attenuated waveform. This evidences an alternative mode of beating—which we conjecture to exist as one of a discrete spectrum of modes on the eukaryotic flagellum. This second mode can also be sustained for much longer durations, and in *both* flagella of a particular phototaxis mutant of *Chlamydomonas*, as detailed elsewhere [7]. Taken together, figure 3 encapsulates the three possible biflagellate ‘gaits’, their differences and similarities, highlighting the need for a quantitative formulation similar to that which we present in this article.

Intrinsic differences between the two *Chlamydomonas* flagella, their construction and actuation, underlie this rich assemblage of biflagellate phenomenology. Past experiments have shown such differences to exist, for example in reactivated cell models of *Chlamydomonas* [9], in which the *trans* flagellum has a higher beat frequency than the *cis*. By contrast, we consider here the *in vivo* case (§3.4); by mechanically inducing deflagellation of either *trans* or *cis* flagellum, we render live wild-type cells uniflagellate. This allows us to compare the intrinsic beating dynamics of *cis* versus *trans* flagella. We found that while *cis*-uniflagellated cells tend to beat with the canonical BS-like mode (BS-mode), *trans*-uniflagellated cells can instead sustain the faster mode of beating associated with the phase slip (aBS-mode) (figure 10*a*). Yet by the time the cell is allowed to recover and the missing flagellum is regrown to full length, both flagella resume the BS-mode of beating once more.

Flagellar tracking has enabled us to acquire true spatial localization of the flagellum throughout its dynamic rhythmicity, complementing recent efforts aiming in this direction [4,10]. The need to know precise waveforms has long been an ambition of historic works, in which manual light-table tracings of *Chlamydomonas* flagella were used to elucidate behaviour of the wild-type [11,12], and later also of flagellar mutants [13]. We hope that the findings and methodologies herein presented shall be of broad interest to physicists and biologists alike.

## 2. Background

### 2.1. The enigmatic flagellum beat

At a more fundamental level, how is beating of a single flagellum or cilium generated, and moreover, how can multi-ciliary arrays spontaneously synchronize? For each of us, or at least for the hair-like appendages lining the epithelial cells of our respiratory tracts, this is indeed an important question. Beating periodically, synchronously, and moreover metachronously, multitudes of these cilia drive extracellular fluid flows which mediate mucociliary clearance. These motile cilia, and their non-motile counterparts, are regulated by complex biochemical networks to perform highly specific functions [14,15]. Mutations and defects in these organelles have been increasingly implicated in many human disorders, including blindness, obesity, cystic kidney and liver disease, hydrocephalus, as well as laterality defects, for example *situs inversus totalis* [16,17]. Mice experiments in which nodal flows are artificially disrupted directly link mechanical flows to positioning of morphogens, which in turn trigger laterality signalling cascades [18].

Across the eukaryotic phylogeny, these slender, propulsion-generating appendages possess a remarkably conserved ultrastructure [19]. In recent decades, causality from structure to function within eukaryotic ciliary/flagellar axonemes has been established using sophisticated molecular genetics tools. For the *Chlamydomonas* in particular, rapid freezing of specimens has made possible the capture of axonemal components in near-physiological states, at molecular-level resolution [20]. *Chlamydomonas* flagella have a well-characterized 9 + 2 structure of microtubule doublets (MTDs), along which are rows of the molecular motor dynein (figure 1). These directional motors generate forces parallel to filamentous MTD tracks, which slide neighbouring MTDs past each other. Anchored to the A-tubule of each MTD by their tail domains, these dyneins detach and reattach onto the next B-tubule, consuming adenosine triphosphate in the stepping process. Different dynein species coexist within the flagellar axoneme, with force generation principally provided by outer dyneins, and modulation of flagellar waveform by the inner dyneins. The central pair is thought to transduce signals via the radial spokes [21]. At approximately every 96 nm, this precise arrangement of dyneins, radial spokes and linkers repeats itself [22]. Periodic elastic linkages between neighbouring MTDs called nexins provide the coupling by which dynein-driven filament sliding produces macroscopic bending of the flagellum, which in turn propels the cell through the fluid. Treatments of axonemes which disrupt dynein domains have shown these nexin linkages to function in a non-Hookean manner to provide the elastic restoring force that resists internal filament shear [23]. More recently, detailed three-dimensional tomographic analysis revealed that nexins are in fact one and the same with the dynein regulatory complex, collectively termed the NDRC [24]. The importance of the NDRC within the functioning axoneme, from those of algae to that of humans, has been highlighted in a recent review [25].

With regard to the flagellum-cycling mechanism, consensus is lacking. Timing control appears to be absent, yet much experimental evidence points to selective, periodic, dynein activation [26]. Both power and recovery strokes of the beat cycle involve active force generation by differentially actuated dyneins. Rhythmic beating of the flagellum may arise from dynamical instabilities of dynein oscillations [27,28] and may be closely coupled to the intrinsic geometry of the axoneme [29,30]. With these unanswered questions in mind, there is thus much incentive to analyse the flagellum beat *in vivo*, as this study seeks to demonstrate.

### 2.2. Molecular origins of *cis*–*trans* difference

As with many species of flagellated algae, motility is essential not just for swimming, but also for cell taxis. For *Chlamydomonas*, its two equal-length flagella, each 10–15 µm long, emerge from an ellipsoidal cell body approximately 5 µm in radius. Each cell has a single eyespot, which functions as a primitive photosensor. Perceived directional light is converted downstream via secondary messenger ions into differential flagellar response and cell turning [22,31,32]. The two anterior basal bodies from which the two flagella protrude are connected to each other via distal striated fibres [33]. The eyespot is assembled *de novo* after each cell division [32], breaking cell bilateral symmetry by its association with the daughter four-membered rootlet (figure 1*a*, D4). In reactivated cell models, the *trans* flagellum in particular has been shown to beat at an intrinsic frequency that is approximately 30–40% higher than that of the *cis* [9]. In §3.4, we reveal that this frequency mismatch, additionally with a discrepancy in beating waveform, is discernible *in vivo* in wild-type cells that we rendered uniflagellated through mechanical deflagellation treatment. Differential phosphorylation of an axonemal docking complex has been suggested to underlie the distinctive *cis*–*trans* beat [34]. In particular, differential *cis–trans* sensitivity to submicromolar Ca^2+^ in cell models and in isolated axonemes [9,35] is consistent with the opposing flagellar modulation necessary for cells to perform phototactic turns [13].

Yet, despite these intrinsic differences, the two flagella maintain good synchrony during free swimming [4,10,11], as well as when held stationary by a micropipette (here). Interflagellar coupling may be provided by the motion of the fluid medium [36,37], by rocking of the cell body [5], or further modulated internally via elastic components through physical connections in the basal region [33]. However, stochastically induced flagellar *phase slips* can appear in otherwise synchronized beating flagella in a distinctive, reproducible manner (figure 3). We find that the propensity to undergo these transient slips [13] can vary significantly even between cells of a clonal population (figure 6).

## 3. Results

### 3.1. Three gaits of biflagellate locomotion

#### 3.1.1. The breaststroke

**Figure 2.**
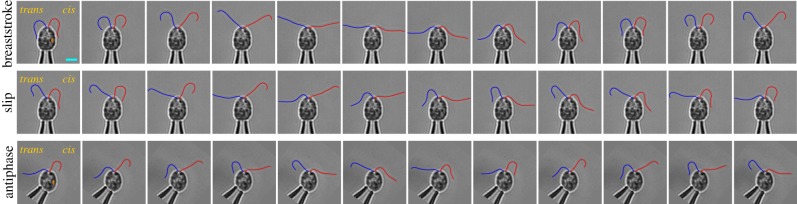
Three *Chlamydomonas* BS swimming gaits recorded at 3000 frames per second and shown at intervals of five frames (i.e. 1.7 ms intervals). Orange dot marks cell eyespot location. Red curves: *cis* flagellum; blue curves: *trans* flagellum. Shown in order, in-phase synchronized BS (both flagella in BS-mode), a phase slip in the same cell (*trans* flagellum in aBS-mode) and antiphase synchronization in the phototaxis mutant *ptx1* (both flagella in aBS-mode). Scale bar, 5 µm.

In their native habitat, *Chlamydomonas* cells swim in water (kinematic viscosity *v* = 10^−6^ m^2^ s^−1^), at speeds of 100–200 µm s^−1^, depending on strain and culture growth conditions [38]. Oscillatory flows set up by their flagella during the BS average frequencies of 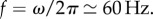 Stroke lengths of *L* = 10 µm produce a tip velocity scale of *U* = *L*ω* ∼ 4 mm s^−1^. An (oscillatory) Reynolds number *Re* = *ω*L*^2^/*v* gauges the viscous and inertial force contributions to the resulting flow. Here, *Re* ≈ 0.001 so that cell propulsion relies on viscous resistance to shearing of the fluid by the flagellar motion. To overcome the reversibility of such flows, a breaking of spatial symmetry is essential during the swimming BS. The rhythmic sweeping motion of each flagellum can be partitioned into distinct power and recovery strokes: during the power stroke, the flagella are extended to maximize interaction with the fluid but are much more curved during the recovery stroke (figure 2). Net swimming progress results from the drag anisotropy of slender filaments and the folding of flagella much closer to the cell body during the recovery stroke. Interestingly, a qualitatively similar bilateral stroke can emerge from a theoretical optimization performed on swimming gaits of biflagellate microorganisms [39] and on single flagella near surfaces [40].

#### 3.1.2. The phase slip

**Figure 3.**
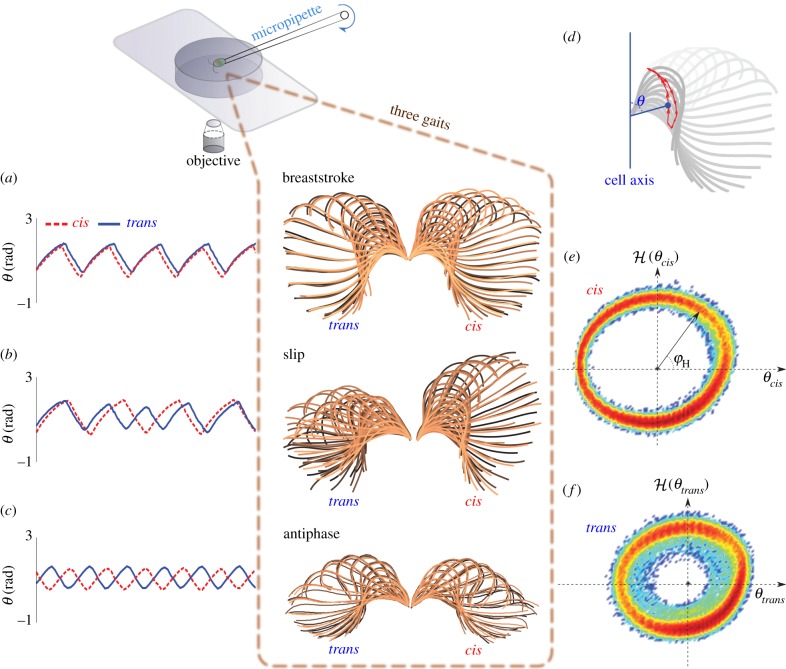
Overlaid sequences of tracked flagella showing (*a*) normal BS (five consecutive beats), (*b*) a stochastic slip event (four consecutive slips) in which the *trans* flagellum transiently enters a different mode (aBS) and (*c*) in which both flagella of *ptx1* sustain the aBS-mode (five consecutive beats). (*d*) For each flagellum, progression through the beat cycle can be tracked using an angle *θ* defined relative to the cell bilateral axis, measured at a fixed arc length along the centre line of the flagellum. Sample time series for *θ* are shown beside the flagellar tracings for each gait. The aBS-mode can clearly be seen to have an attenuated beat amplitude and a faster beat frequency. (*e*,*f*) Differences between *cis* and *trans* limit cycles are clarified in phase-space coordinates 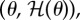 where 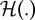 denotes the Hilbert transform (see §3.2). Here, the colour intensity is obtained by logarithmically scaling the probability of recurrence.

Early microscopic analyses of *Chlamydomonas* flagella suggested that the normal BS synchrony ‘may be disturbed for brief periods’ [33]. These interruptions to ‘normal’ beating, subsequently detailed in manual waveform tracings by Rüffer and Nultsch, were shown to occur in the absence of obvious stimulation, in both free-swimming cells [11] and cells affixed to micropipettes [12]. Crucially, these transient asynchronies do not significantly alter the trajectory of swimming [2]; instead, during each such episode the cell body is seen to rock back and forth slightly from frame to frame without altering its prior course. These asynchronies are termed *slips* [2,3] by analogy with an identical phenomenon in weakly coupled phase oscillators. Physically, phase slips are manifest in these coupled flagella in a strikingly reproducible manner. Under our experimental conditions (detailed in §5), beating of the *trans* flagellum transitions during a slip to a distinct waveform, concurrently with an approximately 30% higher frequency [7], while at the same time the *cis* flagellum maintains its original mode of beating throughout, apparently unaffected (figure 3*b*). We find also that the faster, attenuated BS mode (aBS) is sustained by the *trans* flagellum for an integer number of full beat cycles, after which normal synchronized BS resumes.

#### 3.1.3. The antiphase

The aBS waveform assumed by the *trans* flagellum during a slip turns out to be markedly similar to that identified in an anti-synchronous gait displayed by a particular phototaxis mutant of *Chlamydomonas* called *ptx1* [41]. In recent, related work, we make these comparisons more concrete and show that this gait (figure 3*c*) involves the actuation of both flagella in aBS-mode and in precise antiphase with each other [7]. Although the nature of the *ptx1* mutation remains unclear, it is thought that the emergence of this novel gait in the mutant is closely associated with the loss of differential flagellar response to calcium. Indeed, it is the opposing response of the two flagella of wild-type *Chlamydomonas* to elevated Ca^2+^ levels that is thought to underlie its ability to perform phototactic reorientation [22].

### 3.2. Phase dynamics of a single flagellum

Many biological oscillators are spatially extended and are therefore fundamentally high-dimensional dynamical objects, but adopting a phase-reduction approach may facilitate quantitative analyses. In such cases, stable self-sustained oscillations can be represented by dynamics on a limit cycle, which in turn can be parametrized by a monotonically increasing variable called *phase* that represents the instantaneous state of the oscillator.

The natural or interpolated phase *φ*_P_(*t*) of an oscillator is defined to increment linearly between successive crossings of a Poincaré section—a surface everywhere transverse to the flow that represents dynamical trajectories in a space of dimension one less than that of the original phase-space. For discrete, section-dependent marker events {*t_n_*} where trajectories intersect this surface and *t_n_* ≤ *t* ≤ *t_n+_*_1_,3.1
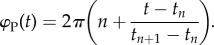


**Figure 4.**
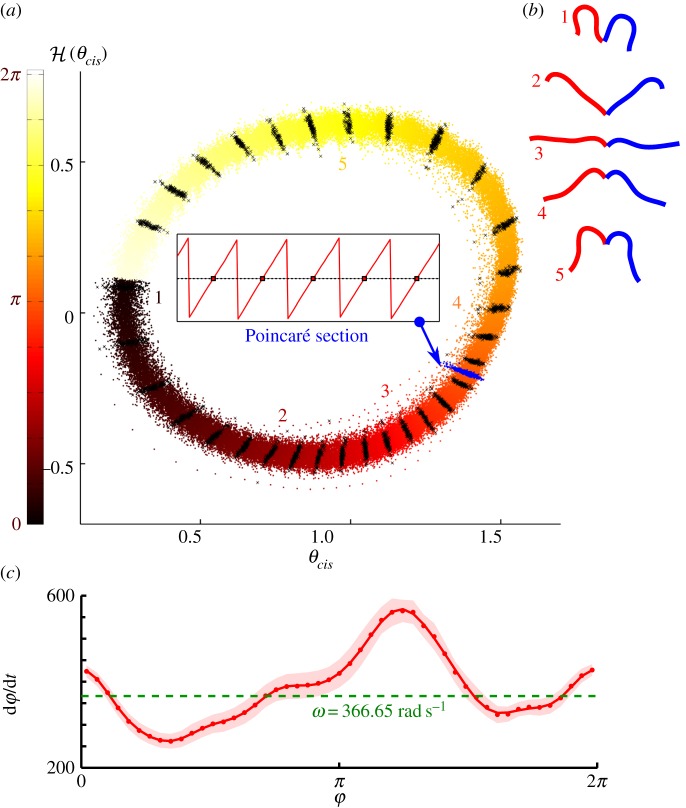
(*a*) Hilbert embedding for the *cis* flagellum of a sample cell from records of 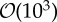 beat cycles and coloured according to the corresponding transformed phase *ϕ* (equation (3.7)). Representative isochrones, or loci of points of equal phase, are highlighted (black lines) at regular intervals of phase. The rate of phase rotation varies systematically throughout the beat cycle, as indicated by the variable inter-isochrone spacing. Inset: temporal marker events defined by successive zero-crossings of 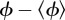 map out one Poincaré section. (*b*) Snapshots 1–5 show typical positions of the flagellum at phases corresponding to five representative isochrones. (*c*) Phase velocity 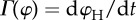 is approximated by a truncated Fourier series. Shaded regions show one standard deviation of fluctuations in the raw data.

This method was used in earlier work [2,3,7] to extract flagellar phases. Pixel intensity variations over pre-defined regions of interest were sampled on individual frames of recorded video; specifically, phase values were interpolated between successive peaks in the measured intensity. However by discretizing via Poincaré sectioning of the dynamics in this way, sub-beat-cycle information is lost. Presently, we adopt a more continuous approach to define phases from two-dimensional projections of flagellum oscillations (figure 4).

For this, we choose an embedding via the Hilbert transform, which derives from the analytic signal concept [42] and is used to unambiguously define an instantaneous phase (and amplitude) from scalar signals with slowly varying frequency [8]. From a periodic scalar time series *x*(*t*), we first construct its complex extension 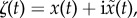 where 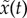 is given by3.2
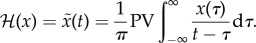
Here, the integral is to be taken in the sense of the Cauchy principal value. The Hilbert phase *φ*_H_(*t*) is given by polar angle rotation in the 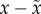 phase-plane,3.3
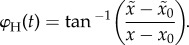
The origin 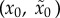 is chosen to be strictly interior of the limit cycle, here, 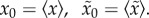


Unwrapping the Hilbert phase *φ*_H_, we obtain a monotonically increasing phase variable on our limit cycle projection. Just as the interpolated phase defined previously, *φ*_H_ does not rotate uniformly and is sensitive to cycle geometry. For any given limit cycle, there is however a unique true phase *ϕ* for which *ω* = d*ϕ*/d*t* = const. (equal to the autonomous oscillator frequency), which is frequently used in theoretical models of interacting oscillators. The rate of rotation of a candidate phase *φ* is in general some non-constant but 2*π*-periodic function *Γ*(*φ*) = d*φ*/d*t*. The transformation3.4
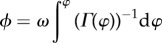
maps *φ* → *ϕ*. While *ϕ* is unique, ambiguity remains in the choice of limit cycle that best characterizes the original phase-space. Furthermore for noisy time series, trajectories do not repeat themselves exactly so that the change of variables equation (3.4) can only be performed in a statistical sense. Accuracy of phase estimation is improved with longer observation time.

To derive *Γ*(*φ*), we first sort data pairs 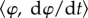 and then average over all ensemble realizations of *φ* (figure 4c). Direct numerical approximations to *Γ*^−1^ = d*t*/d*φ* are sensitive to noise, owing to the heavy-tailed nature of ratio distributions. To remedy this, we follow the approach of Revzen *et al*. [43] and begin by finding an *N*th order truncated Fourier series approximation 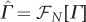 to *Γ*. Next, we find a similar Fourier approximation to 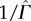
3.5
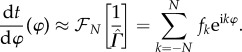
The intrinsic frequency of the oscillator *ω* is related to the zeroth coefficient3.6
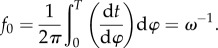
Substituting equation (3.5) into (3.4) and simplifying gives3.7
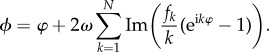
To the lowest order, we see that *φ* and *ϕ* coincide.

For the periodic oscillations of a flagellum, we use the above formulation to derive a phase with which to elucidate within-beat-cycle dynamics. For each flagellum, a limit cycle is constructed from *x* and 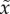 (figure 3*e,f*), where *x* = *θ*(*t*) is the time series of angles subtended at a fixed arc length along the tracked flagellum centre line, relative to a reference axis (figure 3*d*), and 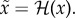 We compute first the Hilbert phase *φ*_H_ along this cycle and use equation (3.7) to associate each data point 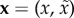 with a unique phase *ϕ*. Points of equal *ϕ* lie on linear structures called isochrones (figure 4*a*); the union of isochrones cover, or foliate, the attracting domain. For different flagella, the function *Γ*(*φ*) measured from experiment takes a characteristic form (figure 4*c*).

**Figure 5.**
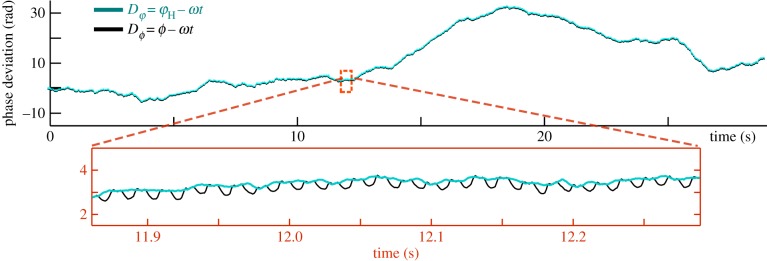
Phase deviation over long time scales (approx. 30 s). *D_*φ*_* and *D_*ϕ*_* are obtained by, respectively, subtracting from unwrapped Hilbert phase *φ*_H_ and transformed phase *ϕ* the linear component that scales with oscillator frequency *ω*. General trends are preserved by the phase transformation (equation (3.4)), but within-cycle fluctuations are removed.

How does the phase of a flagellum fluctuate over long times? In figure 5, we consider two measures of phase deviation: *D*_*ϕ*_ = *ϕ* − *ωt* and *D*_*φ*_ = *φ*_H_ − *ωt* whose long time-scale features coincide, but the periodicity of *D*_*φ*_ has been smoothed out (inset) as perturbations that are 2*π* periodic functions of *φ* are eliminated by the phase transformation [44].

### 3.3. Phase dynamics of coupled flagella pair

For microorganisms that rely on multiple flagella for motility, precision of coordination is essential to elicit high swimming efficacy [39]. The bilateral geometric disposition of the two *Chlamydomonas* flagella facilitates extraction of phases for an individual flagellum's oscillations, and in turn, derivation of phase synchrony relations between the coupled pairs. However, a transformation function similar to equation (3.4) that is bivariate in the two phases cannot be derived from observations of the synchronized state alone. In the following, we make use of the Hilbert phase only (equation (3.3)).

#### 3.3.1. Phase difference derivation

**Figure 6.**
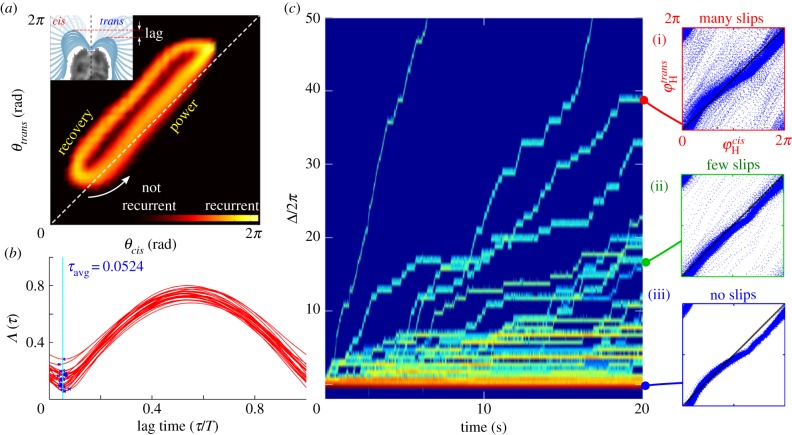
(*a*) Lag synchronization in bivariate time series of flagella beats, shown for a typical cell. Inset: *cis* flagellum begins its recovery stroke fractionally ahead of *trans*. (*b*) Multi-cell similarity functions show a similar trend. Minima cluster around an average (dimensionless) lag time *τ* = 0.0524 ± 0.01, indicative of persistent directional lag. (*c*) Noisy flagellar dynamics within a population of 60 cells, as represented by the tendency of each cell to undergo slips. Most cells remain synchronized for more than 20 s, while some exhibit frequent asynchronies. Red to blue: from high to low probability of recurrence.

**Figure 7.**
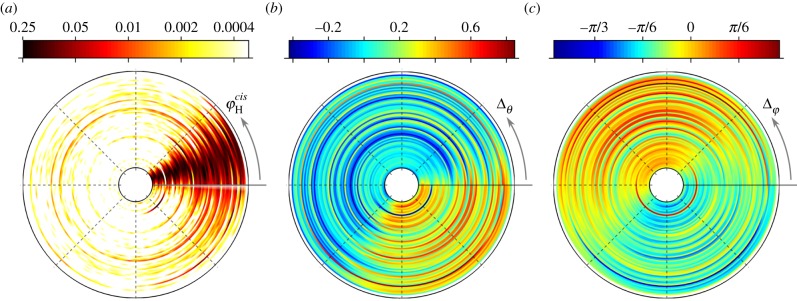
Trends in *cis–trans* flagellar synchronization in a population of *Chlamydomonas* cells: each concentric annulus represents data from an individual cell; measured values are plotted on a circular scale 0 → 2*π* in an anticlockwise sense. Equally spaced radial dotted lines indicate angular progression. (*a*) Probability of stroboscopically observing 
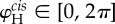
 at discrete marker events where *θ*_*trans*_ reaches a minimum (i.e. start of new power stroke). (*b*,*c*) Difference in tracked angles (Δ_*θ*_ = *θ*_*trans*_ − *θ*_*cis*_) and Hilbert phases 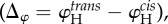 averaged over thousands of beat cycles. It is seen that Δ_*θ*_ is greatest during the recovery stroke, where correspondingly Δ_*φ*_ becomes increasingly negative.

To monitor biflagellar synchrony, the phase difference *φ*_*trans*_ − *φ*_*cis*_ is of particular interest. In general, for coupled noisy phase oscillators *i* and *j*, the dynamics of each is perturbed by the motion of the other, as well as by stochastic contributions:3.8
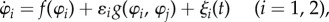
where *g* is a coupling function and 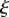 is a noise term. The oscillators are said to be *n:m phase-locked* if their phase difference given by Δ*_n,m_* = *n*φ*_1_ − *m*φ*_2_ is bounded. If instead the system is noisy, phase slips may occur so that strictly speaking Δ*_n,m_* can grow indefinitely. In this case we consider instead the distribution of cyclic relative phase (Δ*_n,m_* mod 2*π*), which will be strongly peaked near the phase-locked value [8]. Here, we define the *trans*–*cis* phase difference from the respective angle signals *θ*_cis_*(*t*) and *θ*_trans_*(*t*) by3.9
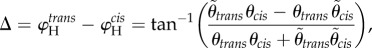
where the tilde again denotes the Hilbert transform. We measured Δ for a large population of cells (figure 6). Phase-slip asynchronies are associated with rapid changes in interflagellar phase difference, and appear as step-like transitions that punctuate (sometimes lengthy) epochs of synchronized behaviour for which Δ = const. We see that over a comparable period of observation time (figure 6*c*(i)–(iii)), pairs of flagella can experience either perfect synchrony, few slips or many slips.^1^ For the population as a whole, the circular representation of figure 7 facilitates simultaneous visualization of general trends in interflagellar phase synchrony.

#### 3.3.2. Lag synchronization

Careful examination of a synchronized epoch shows that Δ is not strictly constant, but rather fluctuates periodically about a constant value. Poincaré sectioning of the dynamics has suggested previously that the BS gait is perfectly synchronized [2,3]. However, plotting *θ*_*cis*_ against *θ*_*trans*_ (figure 6*a*) we see a consistent lag between the two flagella, which is most pronounced during the recovery stroke. By computing and minimizing the similarity function3.10
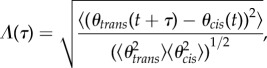
we find this discrepancy to be indicative of lag synchronization. Here, the periodic angle variables *θ*_*cis*__/*trans*_(*t*) were chosen as scalar indicators for the progression of each flagellum through its beat cycle. In particular, the two phases are synchronized with a time lag *τ*_min_ = min*_*τ*_* *Λ*(*τ*), where *Λ*(*τ*) assumes a global minimum. When the oscillators are perfectly synchronized, *τ*_min_ = 0. We calculated *Λ*(*τ*) for multiple cells, which displayed a similar profile (figure 6*b*). With *τ* normalized by the average inter-beat period *T*, in every instance the minimum is fractionally displaced from 0 (or equivalently 1).

#### 3.3.3. Stability of fixed points and transients

The phenomenological model given by equation (3.8) has a convenient dynamical analogy. Phase difference can be interpreted as a particle in a washboard potential 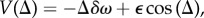 subjected to overdamped dynamics 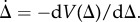 Potential minima occur where 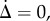 provided 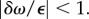 With sufficient noise the particle will have enough energy to overcome the potential barrier. Stochastic jumps between neighbouring potential minima are manifest in coupled flagella as the *slip* mode (figure 3*b*).

In the vicinity of a potential minimum, the stationary distribution of Δ is predicted to be Gaussian (equation (3.8) with white noise). This phase distribution *P*(Δ) can be measured directly from experiment and assumed to satisfy3.11
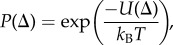
from which the potential structure *U*(Δ) can be recovered. Locations of peaks in *U*(Δ) can be used to estimate the phase lag of the coupled oscillators, while peak widths are proportional to the strength of noise in the system. However with Δ defined as the difference of two (non-uniformly rotating) Hilbert phases (equation (3.9)), we derive an *empirical* potential that is not precisely equivalent to that used in theoretical models of coupled phase oscillators [37].

We measured *P*(Δ) for 18 cells which did not display slips (figure 9*a*). Reconstructed potential minima have a parabolic profile with a well-defined peak on average displaced to the left of Δ = 0, owing to the characteristic lag in the direction of the *cis* flagellum. In certain cells, this lag is especially pronounced during the recovery stroke, resulting in a double-peaked minimum in the fine structure of the empirical potential.

**Figure 8.**
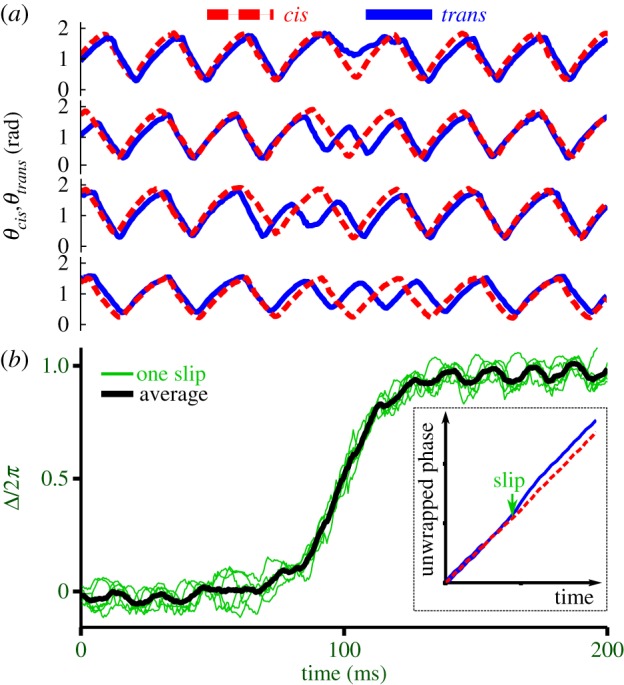
(*a*) Harmonics of a slip. Synchrony resumes after a different (but always integer) number of beats of either flagellum. (*b*) Each slip results in a step-like transition in the phase-difference observable Δ. For the same cell, successive slips overlap. Inset: a single phase-slip event corresponds to forking of unwrapped phases 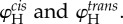

**Figure 9.**
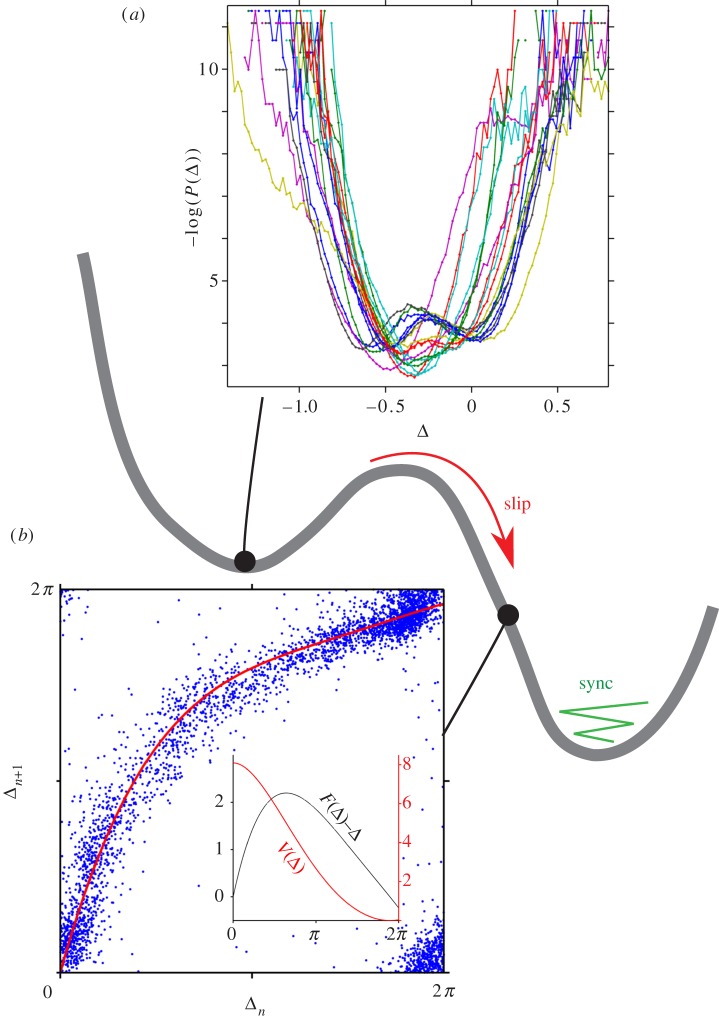
A potential analogy for wrapped phase difference, visualized (*a*) *within* potential minima—Δ exhibits local fluctuations during the stable synchronized gait; and (*b*) *between* potential minima, via a first return map of cyclic/stroboscopic relative phase Δ*_n_*. An *n*th-order polynomial *F*(Δ*_n_*) is fitted to the multi-cell return-map statistics, from which an empirical potential function *V*(Δ) is constructed.

Next, we assess the stability of the synchronized state by observing trajectories that deviate from, but eventually return to, this state; specifically, flagellar phase-slip events are examples of such deviations. They occur with variable duration, and in all cases the *trans* flagellum sustains the faster aBS-mode for a variable but complete number of beat cycles (figure 8*a*). For an individual cell, successive slips often exhibit identical dynamics (figure 8*b*). From multiple slips, we can construct a dimension-reduced return map for the coupled system to visualize the potential landscape that extends between neighbouring minima. We derive a discretized phase difference Δ_*n*_ by evaluating Δ stroboscopically at the position of maximum angular extent of the *trans* flagellum, during the *n*th beat cycle. Using data from more than 500 slip events collected from 70 cells, we plot in figure 9*b* the one-dimensional return map relating Δ_*n*_ to Δ_*n*+1_. Approximating this return map by a polynomial function *F*(Δ_*n*_), stability of the original dynamical system can now be inferred from the stability of the fixed points of *F*. We define an empirical potential function [45] by first integrating the difference 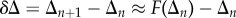:3.12
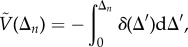
which is made positive-definite via3.13
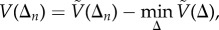
which satisfies 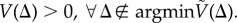 The resulting potential profile (figure 9*b*, inset) represents the reproducible phase dynamics of a typical flagellar phase slip, from which stable BS synchrony re-emerges.

### 3.4. Coupling *cis* and *trans* flagella

Pre-existing, intrinsic differences between the two *Chlamydomonas* flagella are essential for the control of cell reorientation, and loss or reduction in *cis*–*trans* specialization may give rise to defective phototaxis in certain mutants of *Chlamydomonas* [7,46]. Under general experimental conditions, stochastic asynchronies which we call slips can punctuate an otherwise synchronous BS; more drastic loss of interflagellar synchrony can lead to *drifts*, which over time can result in a diffusive random walk in the trajectory of an individual cell [2]. In all these instances, we observe the coupled state of two flagella; by contrast, by mechanically deflagellating wild-type cells (see §5) we now examine the intrinsic behaviour of each oscillator in isolation.

**Figure 10.**
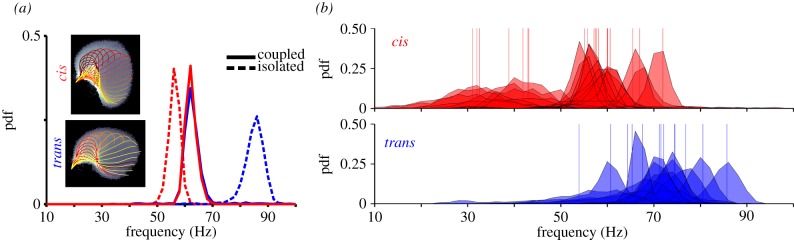
(*a*) For a single cell, *cis* and *trans* flagella were removed and in turn allowed to regrow to full length. Single-flagellum frequencies separate, but once regrown, lock to a common frequency (*cis*: 57.12 Hz, *trans*: 80.52 Hz, both: 63.38 Hz). Insets: typical *cis* and *trans* waveforms. The *cis* and *trans* waveform is reminiscent of the aBS-mode that onsets during a slip. Waveforms are overlaid on an intensity plot of logarithmically scaled residence times for each flagellum [7], over 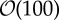 contiguous beats. (*b*) When beating in isolation, *cis* and *trans* flagella have different frequencies: areas, normalized histograms of inter-beat frequencies; lines, averaged frequencies.

The ability of *Chlamydomonas* to readily regenerate a lost flagellum has facilitated controlled measurements of flagellar coupling strength as a function of flagellum length [6]. Using the single eyespot as identifier, we removed either the *cis* or *trans* flagellum from a pipette-captured cell and recorded the beating dynamics of the remaining flagellum. Histograms of beat frequencies are plotted in figure 10*b*. On average, *cis*-uniflagellated cells tend to beat at a lower frequency than *trans*-uniflagellated cells. A dissociation of beat frequency of similar magnitude has been observed previously in reactivated cell models [9]. Moreover, we find that in the absence of the *cis* flagellum the *trans* flagellum can *sustain* the faster aBS-mode for thousands of beats. These differences are highlighted in figure 10*a*, for a single cell.

Interestingly, the aBS-mode that we can now associate with the intrinsic beating waveform of the *trans*, emerges transiently during a slip of the wild-type (figure 3*b*), but in *both* flagella during an antiphase gait of the mutant *ptx1* (figure 3*c*) [7]. Indeed, for *ptx1*, its lack of effectual *cis*–*trans* specialization has led to the speculation that the mutation has rendered both flagella *trans*-like [46]. Specific, structural differences known to exist between the *cis* versus *trans* axonemes [34] of the wild-type may effect this segregation of intrinsic beating modes.

## 4. Discussion

For a unicellular flagellate similar to *Chlamydomonas*, coordination of its flagella is intimately regulated by the cell's internal biochemistry; however, the exact mechanism by which messenger ions modulate and shape the flagellum beat remains unclear. Our experimental technique captures the motion of beating flagella *in vivo*, at high resolution, and with respect to a fixed pivot, thereby permitting long-time analysis.

Associating each flagellum oscillator with a continuous phase, we formulated a phase-reduced model of the periodic dynamics. From long-time-series statistics of bivariate oscillator phases, we used phase difference to track phase synchrony, quantifying flagellar interactions for a single individual as well as across the sampled population. Exquisitely sensitive to its surroundings, a flagellum can be found to undergo precise, yet dynamic changes when executing its periodic strokes. Waveform tracking has allowed us to assess these changes in great spatio-temporal detail.

We have found that the stable phase-locked BS of *C. reinhardtii* exhibits a small but persistent *cis*–*trans* phase lag, whose magnitude and direction were evaluated from statistics of thousands of beat cycles using a similarity measure and confirmed for multiple cells. However, it is not the synchronized state itself but rather the emergence or cessation of synchrony that is most insightful for inferring fluctuations in the physiological state of a complex system. Phase slips are transient excursions from synchrony in which, under our experimental conditions, an alteration of beating mode is observed in the *trans* flagellum only and that appear to be initiated by reduced *cis*–*trans* phase lag (figure 8*b*). These reproducible events highlight the importance of *cis*–*trans* specialization in *Chlamydomonas* flagella. Exploring this further, we mechanically removed individual flagella of wild-type cells to obtain uniflagellated *cis* or *trans* versions, revealing significant differences in their isolated beating behaviours (figure 10). Indeed the loss of one flagellum may induce a transient elevation of Ca^2+^ levels that differentially alters the behaviour of the remaining flagellum, depending on whether it is *cis* or *trans* [9]. It may be that a compromising Ca^2+^ level is maintained in a fully intact cell to minimize these inherent differences between the two flagella, allowing coupling interactions either hydrodynamically through the surrounding fluid medium, and/or biomechanically through elastic linkages at the base of the flagellar protrusion, to enslave the beating of the *trans* mode to that of the *cis*.

Ours is a very versatile technique for quantifying flagellar synchrony not just of the wild-type system, but such a phase analysis can, for instance, also be used to probe defective swimming behaviours of motility mutants. In these cases, macroscopic measurements of population features may not be instructive to understanding or resolving the mutant phenotype and would benefit from dynamic flagellum waveform tracking and in-depth analysis at the level of an individual cell.

## 5. Methods and techniques

### 5.1. Single algal cells on micropipettes

For the purposes of flagella visualization, we chose two wild-type *C. reinhardtii* strains, CC124 and CC125 (Chlamydomonas Center). Stock algae maintained on 2% TAP (Tris-Acetate Phosphate) solid agar slants were remobilized for swimming motility by inoculation into TAP-liquid medium, and cultures used for experimentation were maintained in exponential growth phase (10^5^–10^6^ cells ml^−1^) for optimal motility. Culture flasks were placed onto orbital shakers and maintained at 24°C in growth chambers illuminated on a 14 L : 10 D cycle, so as to imitate the indigenous circadian stimuli. Observation of flagellar dynamics was carried out on a Nikon TE2000-U inverted microscope, at constant brightfield illumination. Additional experiments were also performed with a long-pass filter (622 nm) to minimize cell phototaxis.^2^ Individual cells were captured and held on the end of tapered micropipettes (Sutter Instrument Co. P-97) and repositioned with a precision micromanipulator (Scientifica, UK), and imaged at rates of 1000–3000 frames per second (Photron Fastcam, SA3).

### 5.2. Digital image and signal processing

Recorded movies were transferred to disk for post-processing in Matlab (v. 8.1.0, The Mathworks Inc. 2013). Flagellar waveforms were extracted from individual frames (Image Processing Toolbox), where contiguous dark pixels that localize the moving flagellum were fitted to splines. Hilbert transforms were also performed in Matlab (Signal Processing Toolbox) and further time-series analyses performed using custom Matlab code.

### 5.3. Mechanical deflagellation of either *cis* or *trans* flagella

To obtain the results described in §3.4, individual wild-type cells were first examined under white light to locate the unique eyespot, thereby differentiating its *cis* flagellum from the *trans*. One flagellum was then carefully removed with the second micropipette, by exerting just enough shearing force to induce spontaneous deflagellation by self-scission at the basal region. That cells retain the ability for regrowth of flagella ensures basal bodies have not been damaged by our deflagellation treatment. Cells for which the beating of the remaining flagellum became abnormal or intermittent, and also for which a clear *cis*–*trans* identification could not be made, were duly discarded.

## Endnotes

1 Of the population of cells analysed for figure 6*c*, most were observed under white light. However, a small percentage (10%) were observed under red light, but which for the sake of clarity have not been explicitly marked out in the figure. While in both cases variability in frequency of flagellar slips is observed, we find that on average slips occur more prevalently in cells illuminated by red than by white light (discussed further elsewhere).

2 While cell phototaxis behaviour is minimized under red-light illumination, physiological cell motility cannot be maintained in prolonged absence of light, unless the experimental conditions were accordingly modified [7].

## Data accessibility

Examples of high-speed movies, flagellar time series and other data referenced in this work can be found at http://www.damtp.cam.ac.uk/user/gold/datarequests.html.
